# Importance of TRAIL Molecular Anatomy in Receptor Oligomerization and Signaling. Implications for Cancer Therapy

**DOI:** 10.3390/cancers11040444

**Published:** 2019-03-29

**Authors:** Javier Naval, Diego de Miguel, Ana Gallego-Lleyda, Alberto Anel, Luis Martinez-Lostao

**Affiliations:** 1 Departamento de Bioquímica, Biología Moleculary Celular, Universidad de Zaragoza, 50009 Zaragoza, Spain; diego_demiguel@hotmail.com (D.d.M.); 577565@unizar.es (A.G.-L.); anel@unizar.es (A.A.); 2 Instituto de Investigación Sanitaria de Aragón (ISS), 50009 Zaragoza, Spain; 3 Cell Death, Cancer and Inflammation, University College of London, London WC1E 6BT, UK; 4 Servicio de Inmunología, Hospital Clínico Universitario Lozano Blesa, 50009 Zaragoza, Spain; 5 Departamento de Microbiología, Medicina Preventiva y Salud Pública, Universidad de Zaragoza, 50009 Zaragoza, Spain; 6 Instituto de Nanociencia de Aragón, 50009 Zaragoza, Spain

**Keywords:** TRAIL, oligomerization, clusterization, cancer, immunotherapy, apoptosis

## Abstract

(TNF)-related apoptosis-inducing ligand (TRAIL) is able to activate the extrinsic apoptotic pathway upon binding to DR4/TRAIL-R1 and/or DR5/TRAIL-R2 receptors. Structural data indicate that TRAIL functions as a trimer that can engage three receptor molecules simultaneously, resulting in receptor trimerization and leading to conformational changes in TRAIL receptors. However, receptor conformational changes induced by the binding of TRAIL depend on the molecular form of this death ligand, and not always properly trigger the apoptotic cascade. In fact, TRAIL exhibits a much stronger pro-apoptotic activity when is found as a transmembrane protein than when it occurs as a soluble form and this enhanced biological activity is directly linked to its ability to cluster TRAIL receptors in supra-molecular structures. In this regard, cells involved in tumor immunosurveillance, such as activated human T cells, secrete endogenous TRAIL as a transmembrane protein associated with lipid microvesicles called exosomes upon T-cell reactivation. Consequently, it seems clear that a proper oligomerization of TRAIL receptors, which leads to a strong apoptotic signaling, is crucial for inducing apoptosis in cancer cells upon TRAIL treatment. In this review, the current knowledge of oligomerization status of TRAIL receptors is discussed as well as the implications for cancer treatment when using TRAIL-based therapies.

## 1. Introduction

Apo2 Ligand/TNF-Related Apoptosis Inducing Ligand (Apo2L/TRAIL) was initially described as a TNF family member able to induce apoptosis in a wide range of tumor cells while sparing normal cells [1,2]. This observation lead to the belief that TRAIL could behave as a promising selective anti-tumor agent and in fact, Phase I/II clinical trials were undertaken using TRAIL-based therapeutic agents [3,4]. However, TRAIL-based agents exhibited a limited anti-tumor activity and many primary human tumors were resistant to monotherapy with recombinant soluble TRAIL and other TRAIL receptors agonists [3,5,6]. Since many kinds of drugs may potentiate the TRAIL action in vitro [7], this raises the question whether TRAIL resistance could be overcome by combined treatment with these same drugs. However, recent clinical trials suggest that combination treatments with cytotoxic drugs and TRAIL receptor-targeted agents do not provide additional benefit compared to cytotoxic agents alone [8,9]. It has been suggested that only anticancer drugs able to overcome TRAIL resistance in vitro should be used in vivo [7], such as proteasome inhibitors [7,10], metformin [11], asparaginase [12], or histone-deacetylase inhibitors [13,14]. Anyway, a better anti-tumor effect could be achieved by using improved TRAIL formulations with improved specificity and efficiency.

Many factors contributed to TRAIL resistance and, consequently, to the lack of clinical efficacy of TRAIL-based therapies such as the use of weak TRAIL receptors agonists. In this regard, it has been described that death ligands exhibit a stronger activity when they occur as transmembrane proteins rather than in their soluble form, and this enhanced killing activity is directly linked to their ability to aggregate and arrange their specific receptors in supra-molecular clusters [5]. Hence, the use of novel TRAIL formulations with increased protein stability and cross-linking efficiency seems to be a plausible strategy to improve their clinical success [4,5].

This review summarizes, the current data on the relationship between oligomerization status of TRAIL receptors and bioactivity and the implications for the potential application of TRAIL-based therapy in cancer as well as the main novel TRAIL-based formulations that are currently being developed.

## 2. TRAIL and TRAIL Receptors Structure and Signaling

**Figure 1:**
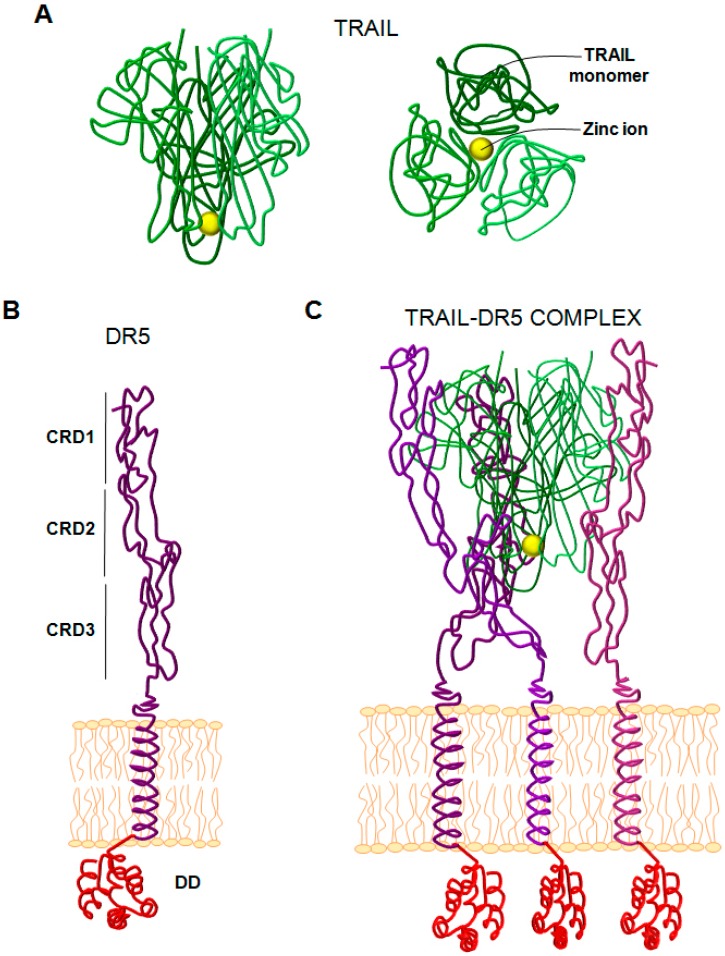
Structure of (TNF)-related apoptosis-inducing ligand (TRAIL) and DR5. (**A**) Structure of the TRAIL trimer. Left panel corresponds to the side view and right panel to the down view. Yellow sphere corresponds to a Zinc ion. (**B**) Structure of the DR5 (TRAIL-R2) monomer anchored to cell surface. CRD (cystein rich domain). DD (death domain). (**C**) TRAIL-DR5 complex. The TRAIL trimer is drawn as tubes rendering in gradations of green, and the three receptor molecules are rendered as tubes in gradations of purple.

Apo2 ligand/TNF-related apoptosis inducing ligand (Apo2L/TRAIL) is a member of the TNF family initially described as capable of inducing apoptosis in a Fas-independent manner [1,2]. TRAIL is a type 2 transmembrane protein encoded by the *TNFSF10* gene located on human chromosome 3 at position 3q26 [15]. The TRAIL monomer consists of a polypeptide moiety of 281 amino acids with a predicted MW of 32.5 kDa, which in its mature, fully glycosylated form, has a MW of 41 kDa. TRAIL presents a potential cleavage site at the extracellular domain (amino acid position 114), which would release a soluble fragment of 24 kDa [1]. The crystal structure of TRAIL reveals a loop formed by an insertion of 12–16 amino acids that penetrates into receptor-binding site, being critical for TRAIL cytotoxic activity [16] (Figure 1). TRAIL forms a homo-trimer that binds three receptor molecules like other TNF family members, so pairs of TRAIL monomers form long crevices that interact with a receptor molecule [16,17]. However, unlike the other TNF family members, TRAIL trimer needs to be stabilized by a Zn^2+^ ion bound to cysteines in the trimer core, which is crucial for the stability, solubility, and bio-activity of TRAIL [18].

TRAIL can bind to four receptors: DR4/TRAIL-R1, DR5/TRAIL-R2, DcR1/TRAIL-R3, and DcR2/TRAIL-R4 [19,20,21,22]. Moreover, a soluble receptor, osteoprotegerin, has been described for TRAIL [23]. Among all these receptors, only TRAIL-R1 and TRAIL-R2 are able to transduce the apoptotic signal, since both DcR1/TRAIL and DcR2/TRAIL-R4 lack functional cytoplasmic death domains (DD) [24,25,26]. In fact, DcR1 and DcR2 have been suggested to act as decoy receptors that inhibit apoptosis induction by TRAIL due to ligand scavenging [26,27]. However, the physiological role of DcR1 and DcR2 is still controversial and their function might depend on the cell type considered.

DR4 and DR5 are type I transmembrane proteins structurally composed of an extracellular region with several cysteine rich domains (CRD), a single transmembrane domain, and an intracellular domain containing the so-called Death Domain (DD) [28,29]. These DDs are homotypic protein interaction modules of about 80 amino acids organized in a bundle of six alpha helices essential for the transduction of the apoptotic signal, acting as binding sites for adaptor proteins [30]. Therefore, the binding of a homotrimer of TRAIL death ligand to three receptor molecules induces a receptor conformational change on the cell surface. Upon this change, the intracellular DD of the three death receptor (DR) molecules undergo a spatial rearrangement, leading to the recruitment of DD-containing adaptor proteins. An adaptor protein called Fas associated death domain (FADD) interacts with TRAIL receptors as well as with Fas [31,32]. The interaction between the corresponding DD of FADD and the TRAIL receptors exposes the death-effector domain (DED) of the apical procaspase-8 or -10. The recruitment of procaspase-8 promotes the assembly of a multi-protein signaling complex called death-inducing signaling complex (DISC), in which procaspase-8 becomes activated by auto-processing followed by its release into the cytosol [30,33]. Finally, active caspase-8 cleaves and activates the effector caspases-3 and -7, which in turn execute the apoptotic cascade [34].

Like other TNF-R family members, TRAIL receptors are physiologically expressed in the cell surface as pre-assembled oligomeric complexes, forming homo-trimers [35,36]. These complexes are formed through interactions of specific extracellular cysteine-rich domains called preligand assembly domains (PLAD), located in the first CRD of the receptor [36]. Formation of PLAD complexes has also been described in the case of Fas [37]. Therefore, a potent pro-apoptotic activity of death ligands is rather dependent on the oligomerization of DR trimers into supramolecular structures [35,38]. This can be only physiologically achieved by high numbers of death ligands expressed in the plasma membrane of effector cells [39] or in the surface of extracellular vesicles [40]. In fact, mutations in the PLAD domain of Fas, which preclude the pre-assembling of trimers, impair apoptotic signaling [37]. Moreover, FasL release in its soluble form due to the action of metalloproteases is a form of functional down-regulation of apoptosis [41,42,43].

## 3. Expression and Secretion of Endogenous TRAIL

Since the major role of TRAIL is the fine-tuning of immune responses [44,45,46], it is not surprising that TRAIL is expressed on a variety of immune cells such as T cells, natural killer (NK) cells, monocytes or dendritic cells.

TRAIL is one of the effector arms of cytotoxic mechanisms of NK cells and plays an important role in suppression of tumor cell growth and prevention of metastasis by NK cells [47,48]. On the other hand, different dendritic cell (DC) subsets express TRAIL. DCs stimulated by interferon-β (IFN-β) express TRAIL on their surface, which is involved in cytotoxicity against tumor cells [49]. IFN-producing killer DCs (IKDCs), which link innate and adaptive immune responses, exhibit cytotoxic activity in part due to the type I IFN-mediated expression of TRAIL [50,51]. Regarding T cells, surface expression of TRAIL can only be detected on activated T cells or monocytes after exposure to IFN-α or IFN-β [52,53,54]. According to this, a low expression of TRAIL is detected in activated T lymphocytes before or after re-stimulation, indicating that it is stored mainly intracellularly before re-stimulation and mainly secreted after re-stimulation [55,56].

Both death ligands, FasL and TRAIL present a cleavage site that releases soluble fragments of 27 kDa and of 24 kDa, respectively. In the case of FasL, the shedding of soluble FasL has been attributed to the metalloprotease ADAM10 [57]. As indicated above, the proteolytic processing of FasL is considered as a mechanism of functional downregulation of apoptosis in healthy cells [43]. In addition, shedding the soluble form of FasL could be a mechanism used by cancer cells to escape from FasL-induced apoptosis exerted by effector immune cells [58,59].

Regarding TRAIL, it has been described that metalloproteinase 2 (MMP2) cleaves the recombinant molecule in vitro [60]. However, no evidence of TRAIL cleavage by metalloproteinases has been found in vivo. Other studies have analyzed the involvement of metalloproteinases in TRAIL cleavage in vitro using the metalloproteinase inhibitor 1,10-phenanthroline. However, this matter still remains controversial since while some authors report that the generation of soluble recombinant TRAIL was not blocked by 1,10-phenanthroline [61], others have described that this metalloprotease inhibitor markedly decreased the production of soluble TRAIL [62]. In fact, in the first study, the authors claimed that soluble recombinant TRAIL generation was dependent on the activity of cysteine proteases instead of metalloproteinases [61].

In any case, although TRAIL and FasL can be secreted as soluble proteins, their physiological mode of action and secretion is as type II transmembrane proteins. Previous work from our group served to characterize the molecular form in which death ligands were released and demonstrated that TRAIL and FasL were rapidly secreted by human activated T lymphocytes undergoing activation-induced cell death in the form of native, non-proteolyzed proteins, associated with a particulate, ultracentrifugable fraction [40]. Characterization of this fraction by scanning electron microscopy showed extracellular vesicles of around 100 nm of diameter that expressed TRAIL and FasL on their surface. Therefore, in a physiological context, both TRAIL and FasL are secreted in their fully native membrane-associated form, keeping their bioactivity intact. 

TRAIL and FasL are stored inside human T cell blasts in a post-Golgi, pre-lysosomal compartment with the structure of a multivesicular body (MVB), mainly associated with intraluminal vesicles [56]. After phytohemagglutinin re-stimulation, human activated T cells secreted these vesicles containing both death ligands, while selective CD59 triggering resulted in the specific release of TRAIL containing vesicles [55]. After re-stimulation of human activated T lymphocytes, the MVB compartments migrated towards the cell membrane, where the external membrane of the MVB eventually becomes fused with the plasma membrane allowing the release of the intraluminal vesicles loaded with bioactive TRAIL and/or FasL [56]. More recently, these extracellular vesicles have been characterized as true exosomes by a combination of proteomic and immunoblotting analysis [63].

## 4. TRAIL in Cancer Immunosurveillance

The role of endogenous TRAIL in tumor immunosurveillance is still not fully understood. Some in vitro studies have demonstrated that NK cells are capable of killing cancer cells by using TRAIL [48,64]. In fact, it seems that TRAIL is involved in the control of tumor metastasis by liver NK cells [65]. Experiments with TRAIL- or TRAIL-R-deficient mice showed that animals were more susceptible to some grafted tumors [66], as well as to chemical carcinogenesis [64,67]. On the other hand, although young mice deficient in TRAIL or TRAIL-R did not exhibit increased tumor incidence [64,68], elder TRAIL-deficient mice showed an increased susceptibility to develop spontaneous lymphoma [69]. This susceptibility seems to be more prominent when there is loss of function of at least one allele of p53 [48,69]. However, other studies did not find an increased rate of spontaneous tumors in TRAIL-R–deficient mice upon loss of p53 [70]. Regardless of these opposing findings in the control of primary tumors, it is commonly accepted that the role of TRAIL in cancer immunosurveillance is mainly devoted to the control of tumor metastasis. This role was initially shown by using grafted syngenic cell lines [47,48,64,71], and later confirmed in a chemically-induced animal model of carcinoma with spontaneous metastasis [72].

On the other hand, although the role of TRAIL in cancer immunosurveillance has been always described as protective, recently, a study describing how endogenous TRAIL/TRAIL-R signaling promotes migration and invasion of KRAS-mutated cancers has been reported. In this context, autocrine activation of DR5 by low levels of endogenous tumor TRAIL triggers activation of Rac1, which in turn, activates phosphatidylinositol 3-kinase to induce cell migration [73]. Interestingly, this activation was found to be independent of the DD, but dependent on the membrane proximal domain of DR5 instead. Finally, endogenous TRAIL induced CCL2 chemokine secretion by TRAIL-resistant tumor cells in a FADD-dependent manner [74]. This TRAIL-induced secretion favors monocyte differentiation to myeloid-derived suppressor cells (MDSCs) and M2-like macrophages, revealing a tumor-supportive immunomodulatory role of TRAIL/TRAIL-R system and therefore outlining a dual role of TRAIL in cancer biology.

## 5. TRAIL and TRAIL Receptors Oligomerization

The structural hallmark of TNF family members is the TNF homology domain (THD), which is part of both the transmembrane and soluble forms of TRAIL. THDs, which are composed of a framework of aromatic and hydrophobic residues, promote self association into trimers mediating receptor binding [75]. However, this interaction alone is not necessarily sufficient to activate receptor-associated intracellular signaling. It seems that after the initial interaction of trimeric ligand with three receptor molecules which are being get together, a multimerization into supramolecular clusters occured [76]. This second step in TNF receptor activation depends on several factors including how the ligand is presented to the receptor, as a membrane, or soluble ligand. In this regard, THD of TRAIL is not sufficient to allow receptor binding and additional spatial fixation and stabilization of their trimeric structure is needed to transmit an apoptotic signal. This fact is reached by the stalk region located on the transmembrane domain, which fixes the N-terminal part of the THD in membrane-bound TRAIL [77]. Therefore, apparently only the transmembrane form of TRAIL, which contains a stalk region, but not soluble TRAIL, can induce supramolecular clusters and elicit a suitable apoptotic signal.

**Figure 2:**
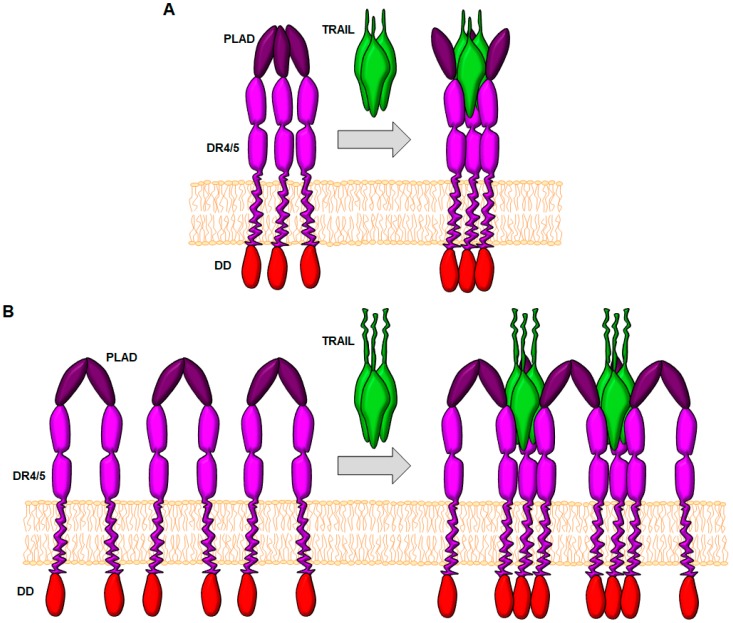
Proposed models for the function of the pre-ligand binding domain (PLAD). (**A**) Model of “conformational change”. In this model, receptors form trimers due to the PLAD, and conformational changes upon TRAIL binding allow juxtaposition of intracellular death domains (DD) of DR4/DR5 receptor molecules. (**B**) Mode of “induction of super-clustering”. In this model, PLAD allows the formation of receptor dimers, and binding of TRAIL promotes a cooperative recruitment of receptors allowing a supra-molecular clustering.

As previously mentioned, TRAIL receptors are physiologically expressed in the cell surface as PLAD similarly to other TNF-R family members [35,36], through interactions of the first CRD of the receptor (Figure 2) [36].

Upon binding of their cognate ligands, the pre-formed oligomers suffer a conformational change that leads to the rearrangement of their intracellular death domains, allowing the recruitment of the DD-containing adaptor proteins FADD and TRADD [31,32], therefore sparking the formation of intracellular protein complexes. In this regard, post-translational modifications such as O-glycosylation could contribute to membrane stability of DRs, preventing endocytosis and facilitating DR translocation into clusters, leading to DISC assembly and caspase-8 activation [78,79].

Death receptors must trimerize in order to trigger the formation of such intracellular complexes, which, as already discussed, can only be achieved by stable trimeric ligands. However, DR5 has a strong tendency to self-association mediated by interactions of the membrane proximal domains. This association in the absence of TRAIL leads to a loss of the threefold symmetry established in the receptor-ligand complex [80]. It seems that TRAIL probably plays a role inducing threefold-symmetry within the DR5 complex and forcing the receptor to a specific conformation. Moreover, the relative amount of the different DRs for the ligand appears to be also important since the formation of non-productive receptor complexes can slow down the formation of active DRs complexes and therefore can block the signal transduction [81]. However, in some cases, the binding of the trimeric ligand is not enough to properly transduce the intracellular signal. In fact, some DRs need to be further cross-linked or oligomerized in high-order clusters on the cell surface to correctly transduce the intracellular signal [82,83,84]. This can only be achieved physiologically by death ligands expressed in high numbers on the plasma membrane of effector cells [39], or on the surface of extracellular vesicles [40].

Apparently, oligomerization of two trimerized receptors would be sufficient to enhance the signaling efficiency, according to different studies [38,84]. Interestingly, other TNF-superfamily members also benefit from this oligomerization effect, such as TNF [85], or FasL [41]. Noteworthy, FasL pro-apoptotic potency is remarkably enhanced upon clustering, reaching a 1000-fold increase [41], and conversely, mutations in the PLAD regions of FAS have been found to participate in pathogenesis of autoimmune lymphoproliferative syndrome [37]. This strongly suggested that DISC was not formed as individual trimers but instead as higher order oligomers. Therefore, once clustered, the receptors would adopt a supra-molecular organization [86]. In this regard, it has been recently reported that the transmembrane helix (TMH) alone in the receptor directly assembles a higher-order structure to drive signaling [87]. Interestingly, the DR5 TMH mediated the generation of multiple trimer-of-dimers that can mediate formation of a patch of dimer-trimer TMH networks on the membrane, forming a “honeycomb” structure. This enhanced aggregation of receptors has proved to cause an improved DISC recruitment [82,86,88]. Although the precise stoichiometry of the DISC is still not completely defined, recent work by Dickens et al. showed that, once activated, trimerized receptors recruit FADD following a receptor:FADD ratio of ≈3:1 in the native TRAIL-induced DISC, which was consistent with the concept of DR5 clusterization. According to this model, caspase-8 would not be only recruited by interacting with FADD, but also with other caspase-8 molecules through homotypic DED interactions, forming elongating “DED chains” [88]. Later studies from the same group also defined the role of other DED-containing proteins such as cFLIP and caspase-10 in the DISC formation and regulation. These data refine the DED-chain model and reinforce the concept of a supra-molecular tridimensional arrangement of DRs as the optimal conformation for an effective DISC formation [89,90].

Remarkably, not all DRs need to be clustered to signal efficiently. For example, TNFR1 can be readily activated by soluble TNF, whilst TNFR2 can only be activated by oligomeric TNF [91]. Similarly, substantial differences have been found between DR4 and DR5. DR5 strictly needs membrane bound/cross-linked TRAIL for signaling whereas DR4 does not [92,93,94]. Interestingly, these differences correlate the localization of both receptors in different cell membrane compartments: apparently, DR4 is required to localize in membrane rafts to be properly activated, while DR5 does not [13,95]. This membrane-raft localization of DR would facilitate clustering and DISC formation just by stimulation with the soluble ligand, whereas DR5, not localized in specific membrane domains, would require to be clustered upon binding to membrane-bound TRAIL.

Considering that death ligands are important mediators of immune signaling, and that they can be physiologically found in both soluble and transmembrane forms, altogether these differences propose an additional level of regulation of the pro-inflammatory and cell death-inducing signaling triggered by the immune system. According to this, metalloproteinase cleavage of membrane-bound FasL, leading to its release as soluble trimers, is a form of functional down-regulation [41,42,43].

On the other hand, it is known that TRAIL receptors form complexes through interactions of PLAD, so that homomeric complexes of DRs promote apoptosis. However, heteromeric assemblies between DRs and DcRs can also occur [96]. In fact, DR4, DcR1, and DcR2, but not DR5, interacted homophilically and heterophilically through their extracellular domains laterally interacting with one another on the cell surface to form pre-assembled complexes prior to ligand binding. These heteromeric interactions could modulate TRAIL-mediated apoptosis and could explain the regulatory role of DcRs in normal cells. In fact, hetero-oligomerization between different TNF receptor superfamily members such as CD40, Fas, and DR5 has been described and could modulate different ligand-induced responses upstream signaling events [97].

## 6. Implication of TRAIL Receptors Oligomerization in Cancer Therapy

TRAIL is considered a promising molecule for cancer treatment for its ability to induce apoptosis in tumor cells without damaging normal cells. This fact has been exploited by research laboratories to develop different TRAIL-based molecules for therapeutic purposes. For an efficient death signal transduction, an optimal receptor aggregation and clustering is needed. Death receptors are expressed on the cell surface as pre-assembled oligomeric complexes whose binding to the ligand provides a conformational change that enables the subsequent binding of adaptor proteins FADD and TRADD via their corresponding intracellular DD [31,32]. However, this is not enough to produce an efficient apoptotic signal transduction. The receptor clustering must occur in high-order supra-molecular structures with a hexagonal organization [82,83,84,88,94,98]. The transmembrane form of ligand (or the cross-linked soluble trimer) is need for an efficient oligomerization, and so, cross-linking ability is a key element for the efficacy of TRAIL-based preparations.

Apoptosis can be induced through each of two functional TRAIL receptors. However, when both receptors are present, DR5 contributes more [99] and is more efficient than DR4 [13] to TRAIL-induced apoptosis in normal and tumor cells. In addition, the level of cross-linking that leads to effective recruitment and activation of the DISC is different between the two receptors. The oligomerization ability of TRAIL-based molecules on DR5 strongly increases the cytotoxic activity in many cells and is often a requirement to obtain an elevated rate of cell death [83]. On the contrary, DR4 activation responds to TRAIL in a cross-linked or non-cross-linked form [93].

Although only recombinant human Apo-2L/TRAIL and agonistic monoclonal antibodies (mAb) specific for DR4 and DR5 have been tested in clinical trials [100,101,102,103,104], many other molecules have been investigated at the preclinical level. We next summarize their most relevant characteristics and the importance of oligomerization.

### 6.1. Recombinant Forms of Human TRAIL

Various recombinant versions of human APO2L/TRAIL have been generated such as leucine-zipper or isoleucine-zipper-tagged TRAIL (LZ-TRAIL or z-TRAIL), which promotes trimerization of the ligand [105,106]. Dulanermin, a recombinant non-tagged TRAIL form, is still the only recombinant protein of Apo2L/TRAIL approved for use in clinical trials [107]. Despite the promising preclinical results, dulanermin did not show a therapeutical benefit. Although the soluble protein can activate apoptosis through DR4 and DR5, other characteristics such as a short half-life or insufficient agonist activity could be decisive in the lack of efficacy. Importantly, dulanermin failed to efficiently achieve clustering of TRAIL receptors, which could most likely explain the poor efficacy shown by this molecule in clinical trials. However, later studies showed that the artificially cross-linked version of dulanermin, called Apo2L.XL, had higher pro-apoptotic ability. This novel form of TRAIL reduced the viability of 257 cell lines out of a panel of 479, while treatment with classic dulanermin affected only to 146 cell lines [108].

#### 6.1.1. Fusion Proteins

The single-chain format of TRAIL (scTRAIL) is a new version generated by the fusion of the three extracellular domains of human TRAIL by peptide linkers to yield highly active single-chain TRAIL. Fusion proteins provide a new approach in TRAIL-based therapies since they allow the generation of oligomerized and optimized trimers, reducing the risk of unspecific aggregation of the monomers. It has been found that the Fn14.TRAIL fusion protein, which combines soluble TRAIL with the TWEAK receptor Fn14, is oligomerized by TWEAK into a super-efficient TRAIL analog. TWEAK is a cytokine frequently found in the tumor environment [109]. The binding of TWEAK to Fn14 provides a further oligomerization of TRAIL receptors that increases their cytotoxic activity. On the other hand, the specificity and targeting of scTRAIL can be improved by fusion to antibody fragments (Fc, Fv) that target TRAIL to specific tumors. Other experimental approach consists in the generation of an agonistic fusion protein, which mimic the natural DR4/DR5-binding sequences of TRAIL fused to the Fc-portion of a human IgG1, a construct called APG350 [110]. The binding of these multimeric constructs to their target subsequently leads to effective clustering of DR5 on cancer cells, therefore, increasing their cytotoxic potential.

These new constructs also better mimic the natural TRAIL expressed on the membrane and are more efficient therapeutics than scTRAIL alone [111]. Moreover, several advantages make this strategy promising for future TRAIL-based therapies. These fusion molecules can activate both TRAIL receptors, thus increasing their cytotoxic activity and, at the same time, the antibody-mediated tumor targeting reduces the potential side effects on healthy tissues. Finally, the possibility of fusing different antibodies to scTRAIL points to the development of personalized treatments for many tumor types [112,113,114,115,116,117].

#### 6.1.2. TRAIL-Mimetic Peptides

Another experimental approach is to use no TRAIL itself but multivalent synthetic peptide agonists of TRAIL using different coupling agents such as adamantane cores [118], adamantane-based dendrons [119], or biomolecular scaffolds of peptidic nature [120]. These multivalent synthetic peptides show an improved affinity for DR5, inducing an enhanced cytotoxic activity due to an increased formation of high-order peptide: receptor complexes (dimeric, trimeric, and hexameric forms). Notably, only multivalent forms of the peptides are able to trigger a significant DR5-dependent apoptotic response, supporting that a copy number of receptor-binding modules is a key prerequisite for proper receptor oligomerization and cell killing. Moreover, these kinds of synthetic drugs took advantage of a high yield production at lower cost and did not have problems related to biological contamination or protein stability during production.

### 6.2. TRAIL Receptors Agonists

**Figure 3:**
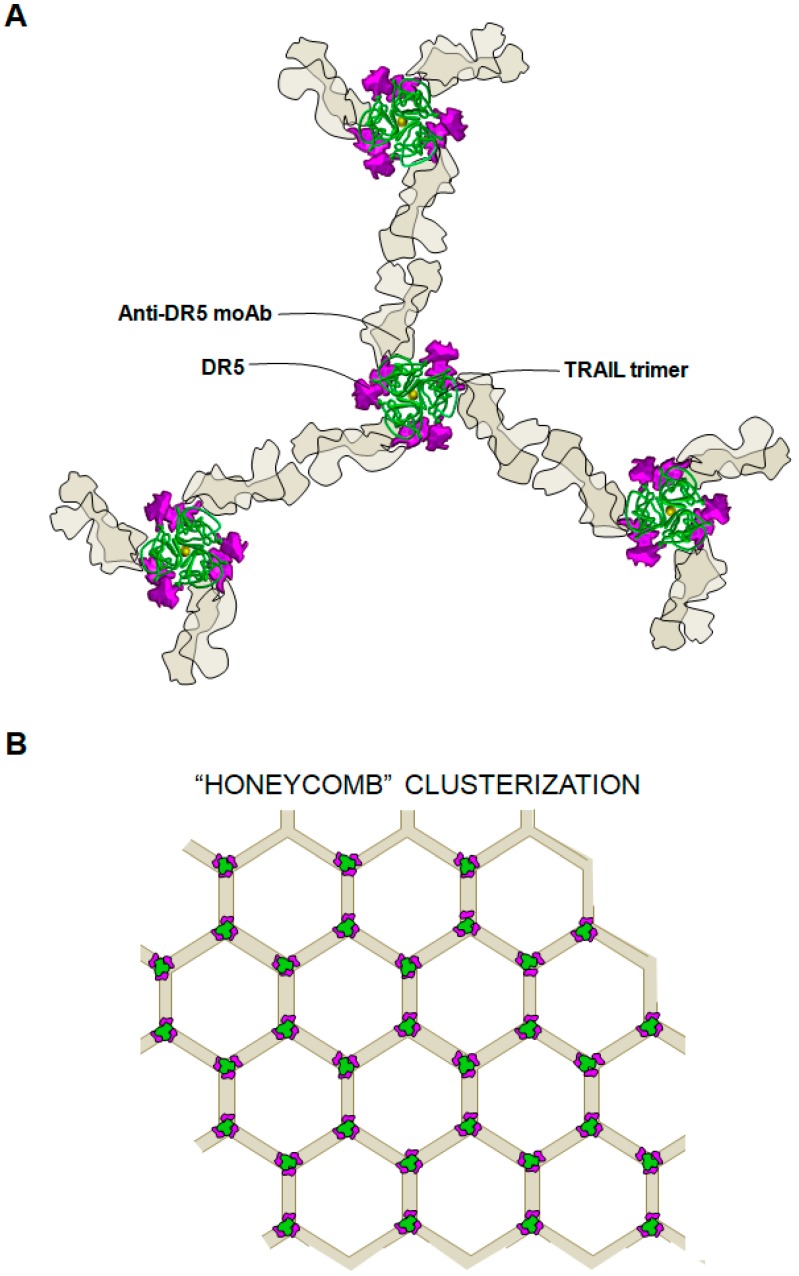
Proposed model of cooperation of TRAIL and anti-DR5 monoclonal antibody to promote receptor clustering. (**A**) View of three TRAIL-anti-DR5 monoclonal antibody ternary complexes (down view). (**B**) Proposed “honeycomb” model of higher order clustering induced by multiple ternary complexes. Green dots correspond to TRAIL bound to three DR5 receptors (purple). Light brown lines correspond to anti-DR5 monoclonal antibody Fab fragments expanding from DR5.

The use of antibody-based therapy has some advantages relative to the soluble form of TRAIL: a longer in vivo half-life, the lack of binding of TRAIL to decoy receptors, and the possibility of the antibody eliciting antibody-dependent cellular cytotoxicity. However, many DR agonistic antibodies require cross-linking for optimal citotoxic activity in vitro and in vivo [121,122,123]. This is the case for drozitumab and AMG655 (conatumumab), antibodies against DR4 and DR5, respectively, which need a further cross-linking to induce apoptosis [122,123]. The FcγR expressed in the membrane of different immune cells physiologically carries this out [124,125]. Although clinical trials have been carried out with antibodies needing cross-linking, such as HGS-ETR2 (lexatumumab) [126], other antibodies possessing improved clustering ability have been developed. For instance, human monoclonal KMTR2 acts as a strong direct agonist for DR5 and is capable of inducing apoptosis without cross-linking [127]. Another strategy recently assayed is the therapeutic combination of agonist anti-TRAIL receptor antibodies and dulanermin. Preclinical studies have shown a synergic effect of an agonistic anti-DR5 mAb (conatumumab) combined with dulanermin in killing primary cancer cells [128,129]. Two independent studies showed that the combination of soluble TRAIL with a specific DR5 agonistic antibody (AMG-655/Conatumumab) could greatly enhance the ability of soluble TRAIL to activate DR5, and even sensitize some otherwise TRAIL-resistant cell lines. This synergistic effect was due to a secondary DR5 cross-linking exerted by the antibody, which enhanced the basal oligomerization of DR5 exerted by soluble TRAIL. This could be done since the epitope recognized by the antibody did not overlap with the surface of interaction of TRAIL with its receptors, therefore not competing with TRAIL-TRAIL receptor interaction. The combination of both agents mimicked the clusterization achieved by membrane-bound TRAIL on DR5, even forming a “honeycomb” structure similar to that observed for FasL-Fas (Figure 3).

### 6.3. TRAIL Anchored to Surfaces

**Figure 4:**
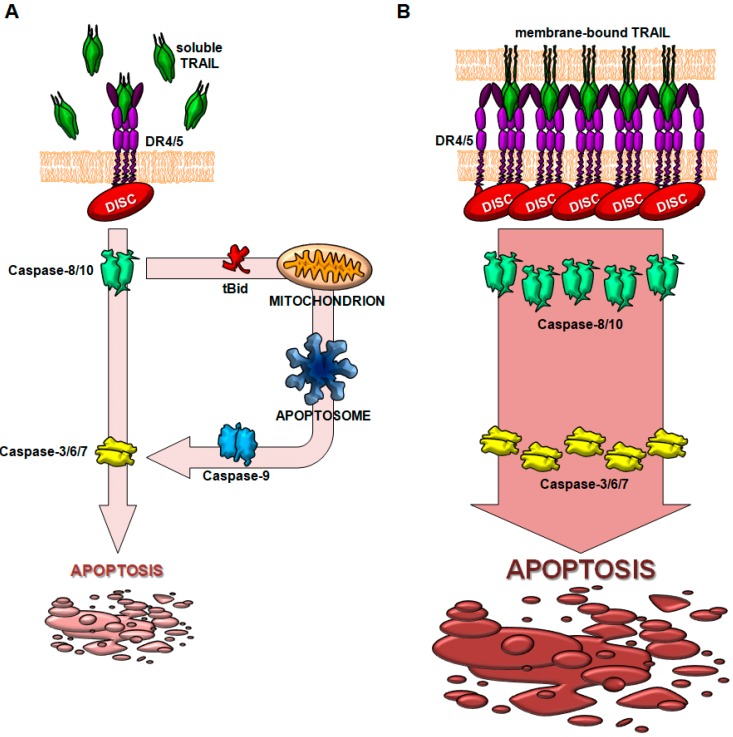
Proposed model for the increase of bioactivity of membrane-bound TRAIL. (**A**) In the case of soluble TRAIL, death receptors (DRs) trimerize and promote the sequential recruitment of FADD and of caspase-8, forming the death-inducing signaling complex (DISC). Caspase-8 processing is enough to trigger apoptosis, but the mitochondrial pathway (through Bid processing) is needed to induce enough caspase-3 activation. (**B**) When TRAIL is present as a membrane-bound protein, TRAIL trimers on the membrane surface induce receptor clusterization and the subsequent enhanced DISC recruitment. Consequently, a high amount of caspase-8 is processed, being enough to directly induce caspase-3 activation.

The proper form of TRAIL expression in cells of the immune system is as a type II membrane protein in the plasma membrane [54,61,130,131,132,133], or inserted in microvesicles [40,55,56]. Whereas DR4 can be activated by the soluble form of TRAIL as well as membrane-bound form of the ligand, DR5 becomes only activated by the membrane-bound form of TRAIL [83]. Interestingly, the restricted ability of soluble TRAIL to induce cell death can be restored by genetic fusion to an antibody derivative. In fact, this strategy allows antigen-dependent immobilization of the fusion protein to cell surface, mimicking a membrane-bound form of TRAIL (Figure 4).

Since TRAIL is physiologically secreted as a membrane protein inserted in exosomes, artificial liposomes with a lipid composition resembling natural exosomes and containing surface-tethered TRAIL have been evaluated in preclinical studies as antitumor agents. Enhanced antitumor activity of this membrane-bound form of TRAIL compared to the soluble form has been demonstrated both in vitro and in vivo against hematological malignancies as well as solid tumors [134,135,136,137,138,139,140,141]. This liposomal formulation with TRAIL anchored to liposome surface induces improved DR5 clustering and enhanced DISC recruitment in comparison to sTRAIL and consequently, a stronger apoptotic signal [135,137]. This superior DR5 clustering and enhanced DISC recruitment is achieved because TRAIL forms high-order TRAIL oligomers on lipid nanoparticle surface [138].

Finally, TRAIL-based nano-vectors have also been designed in order to improve their ability to promote receptor oligomerization [142]. Nanovectorization of TRAIL using single-walled carbon nanotubes mimics membrane-bound TRAIL and significantly increases its anti-tumor activity in vitro, owing to their ability to increase caspase-8 activation.

In conclusion, although soluble TNF ligands, including TRAIL, can interact with their cognate receptors, they poorly activate signaling by these receptors. Therefore, death receptors must at least trimerize in order to trigger the formation of the intracellular complexes necessary to induce the apoptosis. An increased number of evidences suggest that besides the initial formation of trimeric ligand receptor complexes it is necessary a subsequent secondary multimerization into supramolecular clusters. These two sequential steps in death receptor activation, ligand binding, and secondary aggregation of receptor ligand complexes, depend on a several factors from various kinds such as lipid raft localization, receptor auto-aggregation, receptor-associated adapter proteins, post-translational modifications and the affinity and avidity of the ligand for the receptor. Among these factors, it seems crucial the manner in which the ligand is presented to the receptor, either as a transmembrane protein or a soluble protein, as well as in the form of trimers or as high-order aggregates. In this regard, the physiological way of TRAIL secretion, as a transmembrane protein inserted in microvesicles, points to the strategy to reach a proper death receptor clustering allowing the formation of high-order oligomers and the subsequent induction of a strong apoptotic signal. Therefore, the use of membrane-bound or immobilized forms of TRAIL that mimic the transmembrane ligand is key to design biological drugs for cancer therapy in order to achieve optimal apoptotic signaling to efficiently kill cancer cells. Consequently, further studies to deeply understand the molecular anatomy of the interaction of TRAIL with its receptors are necessary to improve TRAIL-based therapies for cancer treatment.
